# Occurrence and challenges in the management of severe chronic plaque type psoriasis in a limited resourced setting: a case report

**DOI:** 10.11604/pamj.2019.32.86.13329

**Published:** 2019-02-20

**Authors:** Calypse Asangbe Ngwasiri, Ngo-Mbaki Charmaine Kwangsa, Martin Hongieh Abanda, Leopold Ndemnge Aminde

**Affiliations:** 1 Clinical Research Education, Networking & Consultancy (CRENC), Douala, Cameroon; 2 Healing Touch Hospital, Muyuka, South-West Region, Cameroon; 3 Banso Baptist Hospital (BBH), Kumbo, Cameroon; 4 School of Public Health, Faculty of Medicine, The University of Queensland, Brisbane, Australia

**Keywords:** Plaque-type psoriasis, indigenous medication, treatment, case report

## Abstract

Plaque-type psoriasis is a major dermatosis with significant effects on quality of life. Case complexity is often high in low-resourced settings such as in Africa where the incidence has been on the rise. Despite major advancements and newer therapeutic modalities over the last decade, an insight into the real-life, day to day challenges in low resourced settings reveal an interplay between the difficulty in obtaining these drugs and use of alternative traditional indigenous agents. We report the case of a 50 year old immunocompetent male who presented with chronic and extensive well demarcated plaques covered with silver-white scales occupying about 61% of his body surface area. Patient was however lost to follow up for about 8 months during which time, the lesions responded to some unknown homemade indigenous medications which was preferred to a systemic medication. Paramount importance on proper counselling and the need to retain patients in care is warranted by physicians and allied health personnel. Also, incentives aimed at subsidizing the newer systemic agents for patients in low resourced cohorts will go a long way to combat this multi-faceted disorder which is often unrecognized and under diagnosed.

## Introduction

Psoriasis is an immune-mediated, complex, chronic, noncontagious multisystem inflammatory disorder that affects approximately 2-3 % of people world. The disease is less common in the tropics and in dark skinned persons with an estimated prevalence of 1.3% in African Americans compared with the 2.5% in whites [1]. There is a huge genetic predisposition for the illness which affects patients far beyond the skin; with joint involvement in about 30% of cases [2]. No specific area limitation is ascribed to disease manifestation and multiple types have been identified, with plaque-type (discoid) psoriasis being the commonest. These plaques appear as well-demarcated, focal, raised edematous lesions covered with silver-white micaceous scales. Diagnosis is mainly clinical, however, a dermatologic biopsy is warranted in those cases where recognition remains a puzzle. Treatment modalities are chosen based on disease severity, relevant comorbidities, patient preference (cost and convenience), efficacy and evaluation of individual patient response [3]. In its severe form, psoriasis can have a remarkable socioeconomic influence as it impacts the affected individual and society at different levels. Various objective and subjective assessments for severity of psoriasis have been discussed; generally relying on scoring systems for which the Psoriasis Area Severity Index score (PASI) is the most adequate available tool [4]. Severe variants require treatment with systemic agents either as monotherapy or combination of multiple agents whose long term safety and laboratory monitoring have to be defined. Recently developed and upcoming biologics offer new therapeutic approaches but the difficulty in reliably obtaining, storing and using them coupled with their high costs is a dilemma for patients with lower socioeconomic status [5]. In low and middle income countries (LMIC), case complexity is often high and available resources from the individual to societal perspective (particularly primary health care) are often suboptimal. We describe herein the case of a 50 year old male in a low-resourced setting with severe chronic plaque-type psoriasis, lost to follow-up in whom management was challenging and difficult; our key objective being to emphasize on the complexity of this multi-system disease in a tropical setting and the need to retain patients in care.

## Patient and observation

**Figure 1:**
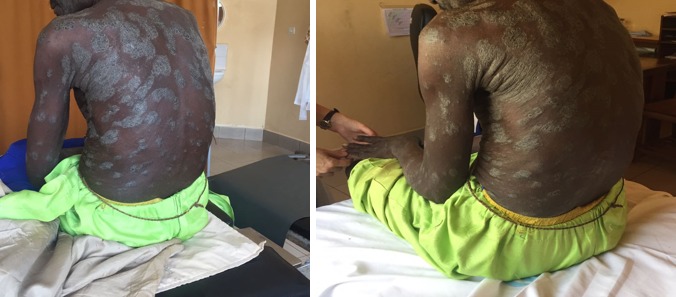
Focal raised inflamed edematous lesions covered with silvery-white micaceous scales

**Figure 2:**
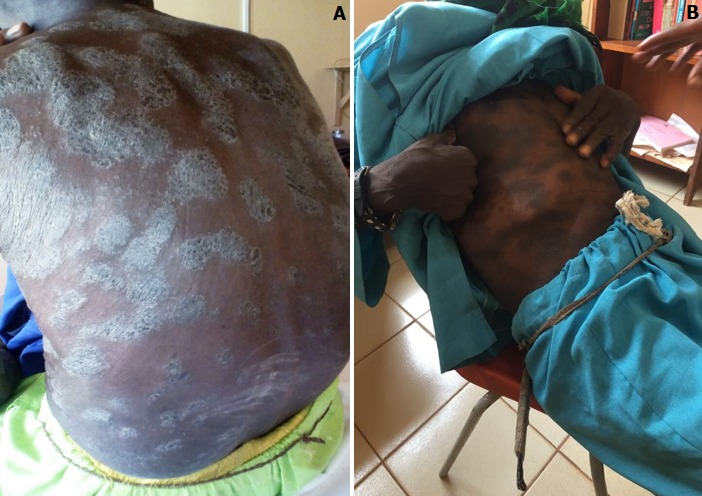
(A) superficial exfoliation of the face and lesions were discrete, firm, and silver grey; (B) resolution of skin eruptions

A 50 year old male cattle herdsman with no known personal or family history of skin disorders presented for evaluation of skin eruptions which had been evolving for about 2 years prior consultation. Patient was living in the forest at the onset of these lesions and noticed they were painless, pruritic and scaly eruptions which developed on his scalp, face and both upper extremities. Initial thought was a supposed fungal infection for which he took both topical and systemic terbinafine (taken liberally as an over the counter drug) for an unknown duration to no benefit. Progress was characterized by worsening of the lesions, with increased size of eruptions, thickening of underlying skin and further involvement of the entire trunk. However, the lower limbs, palms and soles were unaffected. About two weeks prior consultation to our service, he developed red itchy eyes, generalized polyarthralgia, frequent chills and diminished interest in almost all daily activities. He denied previous history of tuberculosis, alcohol consumption and exposure to toxic chemicals. On physical examination, the patient looked well and comfortable with vital signs within normal limits. He had corneal and conjunctival hyperemia with no sign of oedema or cicatricial ectropion. There were well-defined inflammatory plaques covering about 61% of his total body surface area (TBSA) with associated scaling, lichenification and accentuated skin markings (Figure 1). Superficial exfoliation of the face was noted and the lesions were discrete, firm, and silver grey, involving the entire integument but for the lower limbs, palms and soles (Figure 2A).

Nail examination revealed no area of dystrophy or pitting. Examination of the cardiovascular and respiratory systems revealed no abnormality likewise joint examination for any tenderness or edema. Blood and tissue cultures were negative for bacteria, fungi and atypical mycobacteria. Serological tests for HIV and Syphilis were also negative likewise skin snip for filaria. His baseline lipid profile was normal so too was work up for Hepatitis B and C. Punch biopsy of skin was consistent with psoriasis and focal suppurative folliculitis (Microscopic Description: Sections reveal skin with acanthosis, elongated retia, hyperkeratosis with parakeratosis, focal neutrophilic infiltration of the epidermis, loss of granular layer, focal spongiosis and suprapapillary thinning. One fragment showed a suppurative folliculitis with dermal abscess. Dermis with inflammatory infiltrates that include neutrophils is seen in other areas. PAS and Gram stain failed to recover fungal and bacterial organisms). The diagnosis of severe chronic plaque type psoriasis was made based on his psoriasis severity index (PASI) score of 13.6 (> 12) and a BSA 61% (> 10%) (Table 1). Owing to our low income resource, a topical steroid (Betamethasone 0.05% topical), topical moisturizer (petroleum jelly), systemic therapy (methotrexate: 10mg weekly initially) and daily sun exposure were the treatment modalities employed. Fluorometholone Acetate was also added to aid with the ocular inflammation whilst psychiatric evaluation was sort. Patient was however lost to follow up and was seen about 8 months later with complete resolution of plaques following the repeated ingestion of some unknown homemade indigenous medications (Figure 2B) PASI 3.8, which he took in preference to medical treatment.

**Table 1:** Soriasis area and severity index (PASI)

Plaque Characteristics					
		Head	Upper Limb	Trunk	Lower Limb
Erythema		1	1	1	0
Induration/thickness		3	3	3	0
Scaling		4	4	4	0
					
	Lesion Score Sum	8	8	8	0
					
% Affected Area		1	2	4	0
	Subtotal	8	16	32	0
					
Body Surface Area		X 0.1	X 0.2	X 0.3	X 0.4
		0.8	3.2	9.6	0.0

PASI Score = 13.6

PASI< 7; mild chronic plaque-typed psoriasis

PASI 7-12; moderate chronic plaque-typed psoriasis

PASI>12; severe chronic plaque-typed psoriasis

## Discussion

Severe flares of psoriasis can be induced by systemic and environmental factors like life stress events, infections and medications. The most severe forms closely resemble other forms of erythroderma like atopic dermatitis, seborrheic dermatitis, cutaneous T cell lymphoma, mycosis fungoides and pityriasis rubra piliaris both clinically and histologically. Often patient's history and subtle clues in the clinical presentation with skin biopsy specimen aid in the diagnosis in selected situations as was with ours. Although familial incidence is high with adult onset psoriasis (37% having first degree affected family members) [2], it was difficult to ascertain in this patient if there was indeed any family history of psoriasis. The patient had areas of indurated plaques and associated silvery scale on his trunk that would be much more consistent with psoriasis than atopic dermatitis. Furthermore the negative blood tests and the cutaneous histology together ruled out drug induced, atopic, infectious or seborrheic erythroderma. Also, obtaining blood for flow cytometry and Sézary cell count (to assess the potential of blood involvement in cutaneous T cell lymphoma or Sézary syndrome) would've been useful but the absence of atypical lymphocytes on the biopsy specimen made the primary clue for the diagnosis of cutaneous T cell lymphoma unlikely. Pityriasis rubra piliaris is a close differential diagnosis which not uncommonly presents as a diffuse erythroderma with psoriatic-like scales. However, the lack of some clinical distinctive features, such as islands of pale skin (skip lesions), orange-red scaling erythema, and palmoplantar yellowish keratoderma makes psoriasis the more likely diagnosis in our patient. Ocular manifestations are not uncommon in psoriasis with blepharitis being the most common finding. Other manifestations include ectropion and trichiasis, conjunctivitis and conjunctival hyperemia, corneal dryness/melt [6]. Conjunctivitis and conjunctival hyperemia were the identified features in the patient. Once diagnosis is made, treatment is based on the severity of the skin condition which is defined by various objective and subjective assessments for which the PASI is the most adequate instrument available to evaluate severity in plaque type psoriasis [4].

Also there has been a general consensus that involvement of the face, palms/soles or disease that is disabling should be considered as severe. Our patient had a PASI score of 13.6 and 61% BSA affected which defines severe psoriasis, qualifying him for treatment with systemic agents. Traditional systemic agents like retinoids, antimetabolites (methotrexate), immunomodulators (cyclosporine) and TNF inhibitors (etanercept) all improve the clinical symptoms in psoriasis by acting on the main pathways of the psoriatic lesion which can either be used alone or in combination (daily sun exposure, sea bathing, topical moisturizers and relaxation) but concerns lie with their long term safety and efficacy [7]. Biologics (like ustekinumab, secukinumab), are the most recent and effective medications for psoriasis but present a big challenge to patients because aside from the difficulty in reliably obtaining, storing and using them, they are very expensive (30,000-80,000 USD/year) and most insurance companies tend not to approved [5]. In sub-Saharan Africa where about 47% of the population live on less than 0.25 USD/day [8] and where cost of treatment is essentially out of pocket, it represents a significant problem for both physicians and patients. The folic acid antagonist methotrexate (MTX) which is relatively cheaper than at least some of the biologic agents was readily available in our context. It is also effective for the treatment of psoriatic arthritis and nail arthritis. Initial thoughts on the mechanism of action centered around the antiproliferative effects on DNA synthesis in epidermal cells; subsequent evidence supports the concept that it is the immunosuppressive effects of methotrexate on T-cells that controls psoriasis [9]. It was started at an initial weekly dose of 10mg together with adjunctive therapy (phototherapy, sea bathing). Folic acid 1mg daily was also given to protect against common side effects like stomatitis. The patient however deferred medical treatment and was lost to follow-up, preferring some homemade indigenous traditional medication whose beneficial effects resulted in resolution of eruptions about 8 months later. No information about the drug name nor active ingredient could be got. This wasn't entirely surprising as it is well established that some indigenous methods like wet cupping Hijamah and some African traditional plants like Kigelia Africana have been shown to be beneficial in the treatment of psoriasis.

## Conclusion

In our setting where physician inertia and lack of relatively well- equipped dermatology units are common, the appropriate management of severe skin conditions as such remains challenging and difficult. Psoriasis affect patients' perceptions of themselves and this can potentially initiate or exacerbate psychological disorders such as depression, thus proper counselling, empathy and retaining patients in care is absolute. A recent study analyzing skin diseases among elderly patients attending a dermatology unit in Tanzania revealed an overall prevalence of psoriasis to be 7.7%, which is relatively higher than the worldwide prevalence of 2.2-3.9% [10]. This could serve as a wakeup call to raise awareness of the complexity of this multifaceted disease especially as most of the milder cases could go undiagnosed or discovered incidentally. The need for early diagnosis, appropriate referral and comprehensive state-of-the-art approach in the management of patients with psoriasis cannot be overemphasized.

## Competing interests

The authors declare no competing interests.
